# Computation of Kullback–Leibler Divergence in Bayesian Networks

**DOI:** 10.3390/e23091122

**Published:** 2021-08-28

**Authors:** Serafín Moral, Andrés Cano, Manuel Gómez-Olmedo

**Affiliations:** Computer Science and Artificial Intelligent Department, University of Granada, 18071 Granada, Spain; smc@decsai.ugr.es (S.M.); acu@decsai.ugr.es (A.C.)

**Keywords:** probabilistic graphical models, learning algorithms, Kullback–Leibler divergence

## Abstract

Kullback–Leibler divergence KL(p,q) is the standard measure of error when we have a true probability distribution *p* which is approximate with probability distribution *q*. Its efficient computation is essential in many tasks, as in approximate computation or as a measure of error when learning a probability. In high dimensional probabilities, as the ones associated with Bayesian networks, a direct computation can be unfeasible. This paper considers the case of efficiently computing the Kullback–Leibler divergence of two probability distributions, each one of them coming from a different Bayesian network, which might have different structures. The paper is based on an auxiliary deletion algorithm to compute the necessary marginal distributions, but using a cache of operations with potentials in order to reuse past computations whenever they are necessary. The algorithms are tested with Bayesian networks from the *bnlearn* repository. Computer code in *Python* is provided taking as basis *pgmpy*, a library for working with probabilistic graphical models.

## 1. Introduction

When experimentally testing Bayesian network learning algorithms, in most of the cases, the performance is evaluated looking at structural differences between the graphs of the original Bayesian network and the learned one [1], as in the case of using the structural Hamming distance. This measure is used in recent contributions as [2,3,4]. A  study and comparison of the different metrics used to measure the structural differences between two Bayesian networks can be found in [1].

However, in most cases the aim of learning a Bayesian network is to estimate a joint probability for the variables in the problem. In that situation the error of a learning procedure should be computed by measuring the difference between the probability associated with the learned network and the original joint probability distribution. Therefore, it can be useful to estimate a network that is less dense than the original one, but in which parameters can have a more accurate estimation. This is the case of the *Naive Bayes* classifier, which obtains very good results in classification problems, despite the fact that the structure is not the correct one. So, in this situation, structural graphical differences are not a good measure of performance.

The basic measure to determine the divergence between an estimated distribution and a true one is the so-called Kullback–Leibler divergence [5]. Some papers use this way of asserting the quality of a learning procedure as in [6,7,8]. A direct computation of the divergence is unfeasible if the number of variables is high. However, some basic decomposition properties [9] (Theorem 8.5) can be applied to reduce the cost of computation of the divergence. This is the basis of the procedure implemented in the Elvira system [10] which is the one used in [6]. Methods in [7,8] are also based on the same basic decomposition. Kullback–Leibler divergence is not only meaningful for measuring divergence between a learned network and a true one, but also for other tasks, as for example the approximation of a Bayesian network by a simpler one [11,12,13] by removing some of the existing links.

The aim of this work is to improve existing methods for computing Kullback–Leibler divergence in Bayesian networks and to provide a basic algorithm for this task using *Python* and integrated into the *pgmpy* [14] environment. The algorithm implemented in the *Elvira system* [10] is based on carrying out a number of propagation computations in the original true network. The  hypothesis underlying our approach is that there are a lot of computations that are repeated in these propagation algorithms, so what it is done is to determine which are the operations with potentials that are repeated and then storing the results in a cache of operations in order to allow reuse them. The experimental work will show that this is an effective method to improve the efficiency of algorithms, especially in large networks.

The paper is organized as follows: Section 2 is devoted to set the basic framework and to present fundamental results for the Kullback–Leibler divergence computation; Section 3 describes the method implemented in the Elvira system for computing Kullback–Leibler divergence; Section 4 is devoted to describing our proposal based on the cache of operations with potentials; Section 5 contains the experimental setting and the obtained results; finally the conclusions are shown in Section 6.

## 2. Kullback–Leibler Divergence

**Figure 1:**
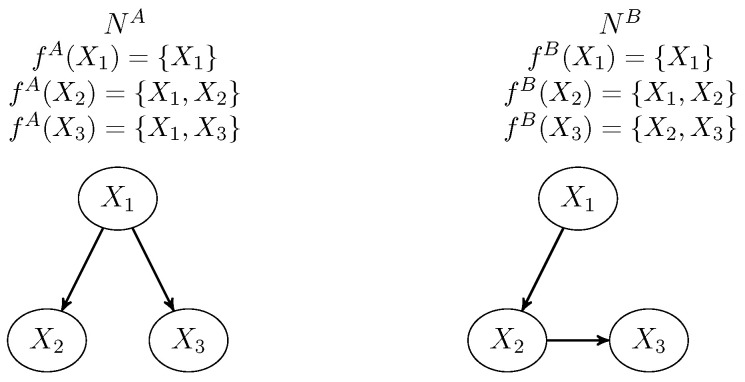
Bayesian networks to compare.

Let *N* be a Bayesian network defined on a set of variables $X=\{X_{1}\ldots X_{n}\}$. The  family of a variable $X_{i}$ in X is termed $f\left(X_{i}\right)=\left\{X_{i}\right\}\cup pa\left(X_{i}\right)$, where $pa(X_{i})$ is the set of parents of $X_{i}$ in the directed acyclic graph (DAG) defined by *N*. $F=\{f\left(X_{1}\right)\ldots f\left(X_{n}\right)\}$ denotes the complete set of families, one for each one of the variables. Sometimes simplified notations for families and parent sets will be used: $f_{i}$ (for $f(X_{i})$) and $pa_{i}$ (for $pa(X_{i})$), respectively. As a running example, assume a network with three variables, $X_{1},X_{2},X_{3}$ and the following structure: $X_{1}\to X_{2}\to X_{3}$ (see right part of Figure 1). Then the set of families for this network is given by $\left\{f_{1},f_{2},f_{3}\right\}$, where $f_{1}=\left\{X_{1}\right\},f_{2}=\left\{X_{2},X_{1}\right\},f_{3}=\left\{X_{3},X_{2}\right\}$.

A configuration or assignment of values to a set of variables X, $\left\{X_{1}=x_{1}\ldots X_{n}=x_{n}\right\}$, can be abbreviated with $\left(x_{1}\ldots x_{n}\right)$ and is denoted as x. If the set of possible values for each variable in the previous example is {0,1}, then a configuration can be x=(0,0,1), representing the assignment $\left\{X_{1}=0,X_{2}=0,X_{3}=1\right\}$.

A partial configuration involving a subset of variables Y⊆X is denoted as y. If the set of variables is $f_{i}$ or $pa_{i}$, then the partial configuration will be denoted by $x_{f_{i}}$ or $x_{pa_{i}}$, respectively. In our example, if $f_{2}=\left\{X_{2},X_{1}\right\}$ an example of partial configuration about these variables will be $x_{f_{2}}=\left(0,0\right)$.

The set of configurations for variables Y is denoted by $\Omega_{Y}$. If x is an assignment and Y⊆X, then the configuration y obtained by deleting the values of the variables in X\Y is denoted by $x^{↓Y}$. If $x_{f_{2}}=\left(0,0\right)$ is a partial configuration about variables $\left\{X_{2},X_{1}\right\}$ and we consider $Y=\{X_{2}\}$, then $x_{f_{2}}^{↓Y}$ is the configuration obtained by removing the value of $X_{1}$, i.e., (0).

If w and z are configurations for W⊆X and Z⊆X respectively, and W∩Z=∅, then (w,z) is a configuration for W∪Z, and will be called the composition of w and z. For example, if w is the configuration (0) about variable $X_{1}$ and y is the configuration (0,1) defined on $X_{2},X_{3}$, then its composition will be the configuration (0,0,1) for variables $\left\{X_{1},X_{2},X_{3}\right\}$.

The conditional probability distribution for $X_{i}$ given its parents will be denoted as $\phi_{i}$ which is a potential defined on the set of variables $f(X_{i})$. In general, a potential ϕ for variables Y⊆X is a mapping defined on $\Omega_{Y}$ into the set of real numbers: $\phi :\Omega_{Y}\to R$. The set of variables of potential ϕ will be denoted as v(ϕ). If Φ is a set of potentials, v(Φ) will denote ${⋃}_{\phi \in \Phi }v\left(\phi \right)$.

In our example, there are three potentials and $\Phi =\{\phi_{1}\left(X_{1}\right),\phi_{2}\left(X_{2},X_{1}\right),\phi_{3}\left(X_{3},X_{2}\right)\}$ (that is, a probability distribution about $X_{1}$, and two conditional probability distributions: one for $X_{2}$ given $X_{1}$ and the other for $X_{3}$ given $X_{2}$, respectively).

There are three basic operations that can be performed on potentials:

- Multiplication. If $\phi ,\phi^{\prime }$ are potentials, then their multiplication is the potential $\phi \cdot \phi^{\prime }$, with set of variables $v\left(\phi \cdot \phi^{\prime }\right)=v\left(\phi \right)\cup v\left(\phi^{\prime }\right)$ and obtained by pointwise multiplication:
  $$\phi \cdot \phi^{\prime }\left(y\right)=\phi \left(y^{↓v(\phi )}\right)\cdot \phi^{\prime }\left(y^{↓v(\phi^{\prime })}\right).$$
  In our example, the combination of $\phi_{2}$ and $\phi_{3}$ will be the potential $\phi_{2}\cdot \phi_{3}$ defined on $\left\{X_{1},X_{2},X_{3}\right\}$ and given by $\phi_{2}\cdot \phi_{3}\left(x_{1},x_{2},x_{3}\right)=\phi_{2}\left(x_{2},x_{1}\right)\cdot \phi_{3}\left(x_{3},x_{2}\right)$.
- Marginalization. If ϕ is a potential defined for variables Y and Z⊆Y, then the marginalization of ϕ on Z is denoted by $\phi^{↓Z}$ and it is obtained by summing in the variables in Y\Z:
  $$\phi^{↓Z}\left(z\right)=\sum_{y^{↓Z}=z}\phi \left(y\right).$$
  When Z is equal to Y minus a variable *W*, then $\phi^{↓Z}$ will be called the result of removing *W* in ϕ and also denoted as $\phi^{-W}$. In the example, a marginalization of $\phi_{3}$ is obtained by removing $X_{3}$ producing $\phi_{3}^{-X_{3}}$ defined on $X_{2}$ and given by $\phi_{3}^{-X_{3}}\left(x_{2}\right)=\phi_{3}\left(0,x_{2}\right)+\phi_{3}\left(1,x_{2}\right)$. If $\phi_{3}\left(x_{3},x_{2}\right)$ represents the conditional probability of $X_{3}=x_{3}$ given $X_{2}=x_{2}$, then it can be obtained that $\phi_{3}^{-X_{3}}\left(x_{2}\right)$ is always equal to 1 ($\forall x_{2}\in v\left(\phi_{3}\right)$).
- Selection. If ϕ is a potential defined for variables Y and z is a configuration for variables Z, then the selection of ϕ for this configuration z is the potential $\phi_{Z=z}$ defined on variables W=Y\Z and given by
  $$\phi_{Z=z}\left(w\right)=\phi \left(w,z^{↓Y}\right).$$
  In this expression $\left(w,z^{↓Y}\right)$ is the composition of configurations w and $z^{↓Y}$ which is a configuration for variables v(ϕ). Going back to the example, assume that we want to perform the selection of $\phi_{3}$ to configuration z=(0,1) for variables $\left\{X_{1},X_{2}\right\}$, then ${\phi_{3}}_{Z=z}$ will be a potential defined for variables $\left\{X_{2},X_{3}\right\}\backslash \left\{X_{1},X_{2}\right\}=\left\{X_{3}\right\}$ given by ${\phi_{3}}_{Z=z}\left(x_{3}\right)=\phi_{3}\left(x_{3},1\right)$, as we are reducing $\phi_{3}\left(X_{3},X_{2}\right)$ to a configuration z in which $X_{2}=1$.

The family of all the conditional distributions is denoted as $\Phi =\{\phi_{1},\ldots ,\phi_{n}\}$. It is well known that given *N* the joint probability distribution of the variables in *N*, *p*, is a potential that decomposes as the product of the potentials included in Φ:

(1) $$p=\prod_{\phi_{i}\in \Phi }\phi_{i}$$

Considering the example, $\Phi =\{\phi_{1},\phi_{2},\phi_{3}\}$ and $p=\phi_{1}\cdot \phi_{2}\cdot \phi_{3}$. The marginal distribution of *p* for a set of variables Y⊆X is equal to $p^{↓Y}$. When Y contains only one variable $X_{i}$, then to simplify the notation, $p^{↓Y}$ will be denoted as $p_{i}$. Sometimes it will be needed to make reference to the Bayesian network containing a potential or family. In these cases we will use a superscript. For example, $f^{A}\left(X_{i}\right)$ and $pa^{A}\left(X_{i}\right)$ refer to the family and parents set of $X_{i}$ in a Bayesian network $N^{A}$ respectively.

The aim of this paper is to compute the Kullback–Leibler divergence (termed KL) between the joint probability distributions, $p^{A}$ and $p^{B}$, of two different Bayesian networks $N^{A}$ and $N^{B}$ defined on the same set of variables X but possibly having different structures. This divergence, denoted as $KL(N^{A},N^{B})$ can be computed considering the probabilities for each configuration x in both distributions as follows:

(2) $$KL\left(N^{A},N^{B}\right)=\sum_{x}p^{A}\left(x\right)\log(\frac{p^{A}\left(x\right)}{p^{B}\left(x\right)})$$

However, the computation of the joint probability distribution may be unfeasible for complex models as the number of configurations x is exponential in the number of variables. If p,q are probability distributions on X then the expected *log likelihood* (LL) of *q* with respect to *p* is:

$$LL\left(p,q\right)=\sum_{x}p\left(x\right)\log\left(q\left(x\right)\right),$$

then, from Equation (2) it is immediate that:

(3) $$\begin{array}{c} KL\left(N^{A},N^{B}\right)=\sum_{x}p^{A}\left(x\right)\log\left(p^{A}\left(x\right)\right)-\sum_{x}p^{A}\left(x\right)\log\left(p^{B}\left(x\right)\right)=\\ LL\left(p^{A},p^{A}\right)-LL\left(p^{A},p^{B}\right)=LL\left(N^{A},N^{A}\right)-LL\left(N^{A},N^{B}\right) \end{array}$$

The probability distribution $p^{B}$ can be decomposed as well as considered in Equation (1). Therefore, the term $LL(N^{A},N^{B})$ in Equation (3) can be obtained as follows considering the families of variables in $N^{B}$ and their corresponding configurations, $x^{↓f_{i}^{B}}$:

(4) $$\begin{array}{c} LL\left(N^{A},N^{B}\right)=\sum_{x}p^{A}\left(x\right)\log\left(p^{B}\left(x\right)\right)=\sum_{x}p^{A}\left(x\right)\log\left(\prod_{X_{i}\in X}\phi_{i}^{B}\left(x^{↓f_{i}^{B}}\right)\right)=\\ \sum_{x}p^{A}\left(x\right)\sum_{X_{i}\in X}\log\left(\phi_{i}^{B}\left(x^{↓f_{i}^{B}}\right)\right) \end{array}$$

Interchanging additions and reorganizing the terms in Equation (4):

(5) $$\begin{array}{c} LL\left(N^{A},N^{B}\right)=\sum_{X_{i}\in X}\sum_{x}p^{A}\left(x\right)\log\left(\phi_{i}^{B}\left(x^{↓f_{i}^{B}}\right)\right)=\\ \sum_{X_{i}\in X}\sum_{x_{f_{i}^{B}}}\log\left(\phi_{i}^{B}\left(x_{f_{i}^{B}}\right)\right)(\sum_{x^{↓f_{i}^{B}}=x_{f_{i}^{B}}}p^{A}\left(x\right))=\\ \sum_{X_{i}\in X}\sum_{x_{f_{i}^{B}}}\log\left(\phi_{i}^{B}\left(x_{f_{i}^{B}}\right)\right){\left(p^{A}\right)}^{↓f_{i}^{B}}\left(x_{f_{i}^{B}}\right) \end{array}$$

Equation (5) implies a decomposition of the term $LL(N^{A},N^{B})$ and, as a consequence of $KL(N^{A},N^{B})$ computation as well. Observe that $\phi_{i}^{B}\left(x_{f_{i}^{B}}\right)$ is the value of the potential $\phi_{i}^{B}$ for a configuration $x_{f_{i}^{B}}$ and can be obtained directly from the potential $\phi_{i}^{B}$ of the Bayesian network $N^{B}$. The main difficulty in Equation (5) consists of the computation of ${\left(p^{A}\right)}^{↓f_{i}^{B}}\left(x_{f_{i}^{B}}\right)$ values, as it is necessary to compute the marginal probability distribution for variables in $f_{i}^{B}$, the family of $X_{i}$ in Bayesian network $N^{B}$, but using the joint probability distribution $p^{A}$ associated with the Bayesian network $N^{A}$.

## 3. Computation with Propagation Algorithms

In this section we introduce the category of inference algorithms based on deletion of variables and then we show how these algorithms can be applied to compute the Kullback–Leibler divergence using Equation (5).

### 3.1. Variable Elimination Algorithms

To compute ${\left(p^{A}\right)}^{↓f_{i}^{B}}$ we consider $\Phi^{A}$ the set of potentials associated to network $N^{A}$: the multiplication of all the potentials in $\Phi^{A}$ is equal to $p^{A}$. Deletion algorithms [15,16], can be applied to $\Phi^{A}$ to determine the required marginalizations. The basic step of these algorithms is the deletion of a variable from a set $\Phi^{A}$:

- **Variable Deletion**. If Φ is a set of potentials, the deletion of $X_{i}$ consists of the following operations:
  – Compute $\Phi_{i}=\left\{\phi \;:\;X_{i}\in v\left(\phi \right)\right\}$, i.e., the set of potentials containing variable $X_{i}$.
  Compute $\phi^{-i}={\left(\prod_{\phi \in \Phi_{i}}\phi \right)}^{-X_{i}}$, i.e., combine all the potentials in $\Phi_{i}$ and remove variable $X_{i}$ by marginalization.
  Update $\Phi \leftarrow \left(\Phi \backslash \Phi_{i}\right)\cup \left\{\phi^{-i}\right\}$, i.e., remove from Φ the potentials containing $X_{i}$ and add the new potential $\phi^{-i}$ which does not contain $X_{i}$.

The main property of the deletion step is the following: starting with $\prod_{\phi \in \Phi }\phi =q$, then after the deletion of $X_{i}$ from Φ, $\prod_{\phi \in \Phi }\phi =q^{-X_{i}}$. It is easy to see that the deletion of a variable $X_{i}$ can be computed just operating with the elements of Φ defined on $X_{i}$.

If Φ is the initial set of potentials of a network *N*, then $p=\prod_{\phi \in \Phi }\phi$. In order to compute the marginalization of *p* on a set of variables Y⊆X, the deletion procedure should be repeated for each variable $X_{i}$ in X\Y. If the marginal probability distribution for variable $X_{k}$ is to be calculated, any variable in X different from $X_{k}$ should be deleted. The order of variable deletion is not meaningful for the final result, but the efficiency may depend on it.

When there are observed variables, Z=z, then a previous step of selection should be carried out: any potential ϕ∈Φ is transformed into $\phi_{Z=z}$. This step will be called *evidence restriction*. After it *q*, the  product of the potentials in Φ is defined for variables in Y=X\Z and its value is q(y)=p(y,z), i.e.,  the joint probability of obtaining this value and the observations. If  a deletion of variables in W is carried out, then the product of the potentials in Φ is the potential defined for variables Y=(X\Z)\W, and satisfies $q\left(y\right)=p^{↓Y\cup Z}\left(y,z\right)$.

When we have observations and we want to compute the marginal on a variable $X_{i}$, it is well known that not all the initial potentials in Φ are relevant. A previous pruning step can be done using the *Bayes-ball* algorithm [17] in order to remove the irrelevant potentials from Φ before restricting to the observations and carrying out the deletion of variables.

### 3.2. Computation of Kullback–Leibler Divergence Using Deletion Algorithms

Our first alternative to compute $LL(N^{A},N^{B})$ is based on using a simple deletion algorithm to compute the values ${\left(p^{A}\right)}^{↓f_{i}^{B}}\left(x_{f_{i}^{B}}\right)$ in Equation (5). The basic steps are:

- Given a specific variable $X_{i}$, we have that $f_{i}^{B}=\left\{X_{i}\right\}\cup pa_{i}^{B}$. Then for each possible configuration $x_{pa_{i}^{B}}$ of the parent variables, we include the observation $pa_{i}^{B}=x_{pa_{i}^{B}}$ and we apply a selection operation to the list of potentials associated with Bayesian network $N^{A}$ by means of evidence restriction. We also apply a pruning of irrelevant variables using the *Bayes-ball* algorithm.
- Then all the variables are deleted except the target variable $X_{i}$. The  potentials in Φ will be all defined for variable $X_{i}$ and their product will be a potential *q* defined for variable $X_{i}$ such that $q\left(x_{i}\right)={\left(p^{A}\right)}^{↓f_{i}^{B}}\left(x_{i},x_{pa_{i}^{B}}\right)$.
- The deletion algorithm is repeated for each variable $X_{i}$ and each configuration of the parent variables $x_{pa_{i}^{B}}$ in Bayesian network $N^{B}$. So, the number of executions of the propagation algorithm in Bayesian network $N^{A}$ is equal to $\sum_{i=1}^{n}\prod_{X_{j}\in pa^{B}\left(X_{i}\right)}n_{j}$, where $n_{j}$ is the number of possible values of $X_{j}$. This is immediate taking into account that $\prod_{X_{j}\in pa^{B}\left(X_{i}\right)}n_{j}$ is the number of possible configurations $x_{pa^{B}\left(X_{i}\right)}$ of variables in $pa^{B}\left(X_{i}\right)$.

Though this method can take advantage of propagation algorithms to compute marginals in a Bayesian network, and it avoids a brute force computation associated with the use of Equation (2), it is quite time consuming when the structure of the involved Bayesian network is complex.
**Algorithm 1** Computation of LL using an evidence propagation algorithm1:**function** LL($N^{A},N^{B}$)2:    sum←0.0                     ▹ sets initial value to sum3:    **for** each $X_{i}$ in $N^{B}$ **do**4:        **for** each $x_{pa_{i}^{B}}$**do**                ▹ configuration of $X_{i}$ parents5:           Let $\Phi^{\prime }$ the set of relevant potentials from Φ                   ▹ Applying *Bayes-ball*6:           Restrict the potentials in $\Phi^{\prime }$ to evidence $pa_{i}^{B}=x_{pa_{i}^{B}}$7:           Delete in $\Phi^{\prime }$ all the variables in $v\left(\Phi \right)\backslash \left\{X_{i}\right\}$8:           Let *q* the product of all the potential in $\Phi^{\prime }$9:           **for** each $x_{i}$ in $\Omega_{X_{i}}$ **do**10:               $sum\leftarrow sum+q\left(x_{i}\right)\log\left(\phi_{i}^{B}\left(x_{i},x_{pa_{i}^{B}}\right)\right)$11:           **end for**12:        **end for**13:    **end for**14:    return sum15:**end function**

Algorithm 1 details the basic steps of the initial proposal for computing the Kullback–Leibler divergence. This algorithm is the one used in [8,10]. Observe that this algorithm computes $LL(N^{A},N^{B})$. It allows the computation of the KL divergence by using Equation (3).

As an example, let us suppose we wish to compute the KL divergence between two Bayesian networks $N^{A}$ and $N^{B}$ defined on $X=\{X_{1},X_{2},X_{3}\}$ (see Figure 1). Let us assume $N^{A}$ is the reference model. The families of variables in both models are presented in Figure 1. We have to compute $LL(N^{A},N^{A})$ and $LL(N^{A},N^{B})$. To compute $LL(N^{A},N^{B})$ Algorithm 1 is applied. Initially, $\Phi =\{\phi_{1}^{A},\phi_{2}^{A},\phi_{3}^{A}\}$ where $\phi_{i}^{A}$ is defined for variables $f^{A}\left(X_{i}\right)$. The algorithm works as follows:

- The parent set for $X_{1}$ is empty. The set of relevant potentials in network $N^{A}$ to compute the marginal for $X_{1}$ is given by $\Phi^{\prime }=\left\{\phi_{1}^{A}\right\}$, which is the desired marginal *q*.
- The parents set for $X_{2}$ in $N^{B}$ is $\left\{X_{1}\right\}$. So, for  each value $X_{1}=x_{1}$ we have to introduce this evidence in network $N^{A}$ and compute the marginal on $X_{2}$. The set relevant potentials is $\Phi^{\prime }=\left\{\phi_{1}^{A},\phi_{2}^{A}\right\}$. These potentials are reduced by selection on configuration $X_{1}=x_{1}$. If we call $\phi_{4},\phi_{5}$ the results of reducing $\phi_{1}^{A},\phi_{2}^{A}$, respectively, then $\phi_{4}$ is a potential defined for the empty set of variables and determined by its value $\phi_{4}\left(\right)$ for the empty configuration. $\phi_{5}$ is a potential defined for variable $X_{2}$. The desired marginal *q* is the multiplication of these potentials: $q\left(x_{2}\right)=\phi_{4}\left(\right)\cdot \phi_{5}\left(x_{2}\right)$.
- The parents set for $X_{3}$ in $N^{B}$ is $\left\{X_{2}\right\}$. So, for each value $X_{2}=x_{2}$ we have to introduce this evidence in network $N^{A}$ and compute the marginal on $X_{3}$. In this case, all the potentials are relevant $\Phi^{\prime }=\left\{\phi_{1}^{A},\phi_{2}^{A},\phi_{3}^{A}\right\}$. The first step introduces the evidence $X_{2}=x_{2}$ in all the potentials containing this variable. Only $\phi_{2}^{A}$ contains $X_{2}$; therefore the selection ${\phi_{2}^{A}}_{X_{2}=x_{2}}$ is a potential defined on variable $X_{1}$ which we will denote as $\phi_{6}$. So, after that $\Phi^{\prime }=\left\{\phi_{1}^{A},,\phi_{3}^{A},\phi_{6}\right\}$. To compute the marginal on $X_{3}$, we have to delete variable $X_{1}$. As all the potentials in $\Phi^{\prime }$ contains this variable they must be combined for removing $X_{1}$ afterwards, i.e.,  computing ${\left(\phi_{1}^{A}\cdot \phi_{3}^{A}\cdot \phi_{6}\right)}^{-X_{1}}$. After this operation, this will be the only potential in $\Phi^{\prime }$ and it is the desired marginal *q*.

## 4. Inference with Operations Cache

The approach proposed in this paper is based on the following fact: the computation of KL divergence using Equations (3) and (5) requires us to obtain the following families of marginal distributions:

- ${\left(p^{A}\right)}^{↓f_{i}^{B}}$, for each $X_{i}$ in $N^{B}$, for computing $LL(N^{A},N^{B})$
- ${\left(p^{A}\right)}^{↓f_{i}^{A}}$, for each $X_{i}$ in $N^{A}$, for obtaining $LL(N^{A},N^{A})$

We have designed a procedure to compute each one of the required marginals ${\left(p^{A}\right)}^{↓Y}$ for each $Y\in \left\{f_{i}^{A}\;:\;X_{i}\in N^{A}\right\}\cup \left\{f_{i}^{B}\;:\;X_{i}\in N^{B}\right\}$. Marginals are computed by deleting the variables not in Y. The procedure uses a cache of computations which can be reused in the different marginalizations in order to avoid repeated computations.

We have implemented a general procedure to calculate the marginal for a family Y of subsets Y of X in a Bayesian network *N*. In  our case the family Y is ${\left(p^{A}\right)}^{↓Y}$ for each $Y\in \left\{f_{i}^{A}\;:\;X_{i}\in N^{A}\right\}\cup \left\{f_{i}^{B}\;:\;X_{i}\in N^{B}\right\}$ and the Bayesian network is $N^{A}$. A previous step consists of determining the relevant potentials for computing the marginal on a subset Y, as  not all the initial potentials are necessary. If Φ is the list of potentials, then a conditional probability potential $\phi_{i}$ for variable $X_{i}$ is relevant for Y when $X_{i}$ is an ascendant for some of the variables in Y. This is a consequence of known relevance properties in Bayesian networks [17]. Let us call $\Phi_{Y}$ the family of relevant potentials for subset Y.

Our algorithm assumes that the subsets in Y are numbered from 1 to *K*: $\left\{Y_{1},\ldots ,Y_{K}\right\}$. The algorithm first carries out the deletion algorithm symbolically, without actually doing numerical computations, in order to determine which of them can be reused. A symbolic combination of ϕ and $\phi^{\prime }$ consists of determining a potential $\phi \cdot \phi^{\prime }$ defined for variables $v\left(\phi \right)\cup v\left(\phi^{\prime }\right)$ but without computing its numerical values (only the scope of the resulting potential is actually computed). This procedure is analogously done in the case of marginalization.

In fact, two repositories are employed: one for potentials ($R_{\Phi }$) and another for operations ($R_{O}$). The entry for each potential in $R_{\Phi }$ contains a value acting as its identifier (id); the potential itself; the identifier of the last operation for which this potential was required (this is denoted as potential time). Initially, $R_{\Phi }$ contains the potentials in Φ assigning time=0 to all of them. When a potential is no longer required, then it is removed from $R_{\Phi }$ in order to alleviate memory space requirements. The potentials representing the required marginals (the results of the queries) are set with time=−1 in order to avoid their deletion.

The repository $R_{O}$ contains an entry for each operation (combination or marginalization) with potentials performed during the running of the algorithm in order to compute the required marginals. This allows that if an operation is needed in the future, its result can be retrieved from $R_{O}$ preventing repeated computations. Initially $R_{O}$ will be empty. At the end of the analysis, it will include the description of the elementary operations carried out throughout the evaluation of all the queries. Two kinds of operations will be stored in $R_{O}$:

- combination of two potentials $\phi_{1}$ and $\phi_{2}$ producing a new one as result, $\phi_{r}$.
- marginalization of a potential $\phi_{1}$, in order to sum-out a variable and obtaining $\phi_{r}$ as result.

The operation description will be stored as registers $\left(id,type,\phi_{1},\phi_{2},\phi_{r}\right)$ with the following information:

- A unique identifier for the operation (id; an integer).
- The type of operation (type): marginalization or combination.
- Identifiers of the potentials involved as operands and result (identifiers allow to retrieve potentials from $R_{\Phi }$). If the operation is a marginalization, then $\phi_{2}$ will identify the index of the variable to remove.

The computation of a marginal for a set Y will also require a deletion order of variables in some cases. This order is always obtained with a fixed triangulation heuristic min−weight [18]. However, the procedure described here does not depend on this heuristic and any one of them could be used.

Algorithm 2 depicts the basic structure of the procedure. The result is LR, an ordered list of *K* potentials containing the required marginals for $Y_{1},\ldots ,Y_{K}$. The algorithm is divided into two main parts.

In the first part (lines 2–26), the operations are planned (using symbolic propagation) and detecting repeated operations. It is assumed that there are two basic functions SCombine$\left(\phi_{1},\phi_{2}\right)$ and SMarginalize(ϕ,i), representing the symbolic operations: SCombine$\left(\phi_{1},\phi_{2}\right)$ will create a new potential $\phi_{r}$ with $v\left(\phi_{r}\right)=v\left(\phi_{1}\right)\cup v\left(\phi_{2}\right)$ and SMarginalize(ϕ,i) producing another potential $\phi_{r}$ with $v\left(\phi_{r}\right)=v\left(\phi \right)\backslash \left\{X_{i}\right\}$.

We will also consider that there are two conditional versions of these operations: if the operation already exists, only the time is updated, and if it does not exist it is symbolically carried out and added to the repository of operations. The conditional combination will be CondSCombine$\left(\phi_{1},\phi_{2},t\right)$ and the conditional marginalization will be CondSMarginalize(ϕ,i) and are depicted in Algorithms 3 and 4, respectively. It is assumed that both repositories are global variables for all the procedures. The potentials representing the required marginals are never deleted. For that, a time equal to −1 is assigned: if time=−1, then the potential should not be removed and then this time is never updated. We will assume the function UpdateTime(ϕ,t) which does nothing if the time of ϕ is equal to −1, and updates the time of ϕ to *t* otherwise in repository $R_{\Phi }$.

Observe that the first part of Algorithm 2 (lines 2–26) just determines the necessary operations for the deletion algorithm for for all the marginals, while the second part (lines 27–32) carries out the numerical computations in the order that was established in the first part. After each operation, the potentials that are no longer necessary are removed from $R_{\Phi }$ and their memory is deallocated. We will assume a function DeleteIf(ϕ,t) doing this (remove from $R_{\Phi }$ if time of ϕ is equal to *t*).

As mentioned above, the analysis of the operation sequence will be carried out using symbolic operations and taking into account the scopes of potentials but without numerical computations. This allows an efficient analysis. The result of the analysis will be used as an operation planning for the posterior numerical computation.

Assume the same example considered in previous sections for the networks in Figure 1. The marginals to compute on $N^{A}$ (as reference model) will correspond to families $f^{A}\left(X_{1}\right)=\left\{X_{1}\right\}$, $f^{A}\left(X_{2}\right)=\left\{X_{1},X_{2}\right\}$, $f^{A}\left(X_{3}\right)=\left\{X_{1},X_{3}\right\}$ and $f^{B}\left(X_{3}\right)=\left\{X_{2},X_{3}\right\}$ (observe that $f^{A}\left(X_{1}\right)=f^{B}\left(X_{1}\right)$, and  $f^{A}\left(X_{2}\right)=f^{B}\left(X_{2}\right)$). Therefore, in this case $Y=\{\left\{X_{1}\right\},\left\{X_{1},X_{2}\right\},\left\{X_{1},X_{3}\right\},\left\{X_{2},X_{3}\right\}\}$.

Initially, the potentials repository $R_{\Phi }$ contains the potentials of $N^{A}$ (a conditional probability for each variable given its parents): $\phi_{1}^{A}\left(X_{1}\right)$, $\phi_{2}^{A}\left(X_{2},X_{1}\right)$, and $\phi_{3}^{A}\left(X_{3},X_{1}\right)$ with time 0. We indicate the variables involved in each potential. The  operations repository, $R_{O}$, will be empty. Table 1 contains the initial repositories. Notice that the superscript *A* has been omitted in order to simplify the notation.
**Algorithm 2** Computation of marginals of *p* for subsets Y∈Y
1:**function** Marginal(N,Y)2:    t←13:    **for** each k=1,…,K **do**4:        Let Y the subset $Y_{k}$ in Y5:        Let $\Phi_{Y}$ the family of potentials from Φ relevant to subset Y6:        **for** $X_{i}\in v\left(\Phi_{Y}\right)\backslash Y$ **do**             ▹ determine operations for the query7:           Let $\Phi_{i}=\left\{\phi \in \Phi_{Y}\;:\;X_{i}\in v\left(\phi \right)\right\}$8:           Assume $\Phi_{i}=\left\{\phi_{1},\ldots ,\phi_{L}\right\}$9:           $\psi =\phi_{1}$10:           **for** l=2,…,L **do**11:               ψ← CondSCombine$\left(\psi ,\phi_{l},t\right)$12:               t←t+113:           **end for**14:           ψ← CondSMarginalize(ψ,i,t)15:           t←t+116:           $\Phi_{Y}\leftarrow \left(\Phi_{Y}\backslash \Phi_{i}\right)\cup \left\{\psi \right\}$17:        **end for**18:        Assume $\Phi_{Y}=\left\{\phi_{1},\ldots ,\phi_{J}\right\}$              ▹ compute joint distribution19:        $\psi_{k}\leftarrow \phi_{1}$20:        **for** j=2,…,J **do**21:           $\psi_{k}\leftarrow$ CondSCombine$\left(\psi_{k},\phi_{j},t\right)$22:           t←t+123:        **end for**24:        Append $\psi_{k}$ to LR25:        Set time of $\psi_{k}$ to −126:    **end for**27:    T←t−128:    **for** each t=1,…,T **do**  ▹ start numerical computation using operations planning29:        Select register with time *t* from $R_{O}$: $\left(t,type,\phi_{1},\phi_{2},\phi_{r}\right)$30:        Compute numerical values of $\phi_{r}$              ▹ Actual computation31:        DeleteIf$\left(\phi_{1},t\right)$, DeleteIf$\left(\phi_{2},t\right)$, DeleteIf$\left(\phi_{r},t\right)$32:    **end for**33:    **return** LR34:**end function**

**Algorithm 3** Conditional symbolic combination
1:**function** CondSCombine($\phi_{1},\phi_{2},t$)2:    **if** register $\left(id,comb,\phi_{1},\phi_{2},\phi_{r}\right)$ is in $R_{O}$ **then**3:        UpdateTime$\left(\phi_{1},t\right),$ UpdateTime$\left(\phi_{2},t\right),$ UpdateTime$\left(\phi_{r},t\right)$4:    **else**5:        $\phi_{r}=$ SCombine$\left(\phi_{1},\phi_{2}\right)$6:        Add register $\left(id,comb,\phi_{1},\phi_{2},\phi_{r}\right)$ to $R_{O}$ with id as identifier7:        UpdateTime$\left(\phi_{1},t\right),$ UpdateTime$\left(\phi_{2},t\right)$8:        Add $\phi_{r}$ to $R_{\phi }$ with time=t9:    **end if**10:    **return** $\phi_{r}$11:
**end function**



**Algorithm 4** Conditional symbolic marginalization
1:**function** CondSMarginalize(ϕ,i,t)2:    **if** register $\left(id,marg,\phi ,i,\phi_{r}\right)$ is in $R_{O}$ **then**3:        UpdateTime(ϕ,t), UpdateTime$\left(\phi_{r},t\right)$4:    **else**5:        $\phi_{r}=$ SMarginalize(ϕ,i)6:        Add register $\left(id,marg,\phi ,i,\phi_{r}\right)$ to $R_{O}$ with id as identifier7:        UpdateTime(ϕ,t)8:        Add $\phi_{r}$ to $R_{\phi }$ with *t* as time9:    **end if**10:    **return** $\phi_{r}$11:
**end function**



The first marginal to compute is for $Y=\{X_{1}\}$. In this case, the set of relevant potentials is $\Phi_{Y}=\left\{\phi_{1}\right\}$ and there are not operations to carry out. Therefore the first marginal is $\Psi_{1}=\phi_{1}$ which is appended to LR.

The second marginal to be computed is for $Y=\{X_{1},X_{2}\}$. In this case, the relevant potentials are $\Phi_{Y}=\left\{\phi_{1},\phi_{2}\right\}$ and there are no variables to remove, but it is necessary to carry out the symbolic combination of $\phi_{1}$ and $\phi_{2}$ in order to compute $\Psi_{2}$ (lines 19–23 of Algorithm 2). If we call $\phi_{4}$ the result, then the repositories after this operation will be as shown in Table 2.

The third marginal to compute is for set $Y=\{X_{1},X_{3}\}$. Now, the relevant potentials are $\Phi_{Y}=\left\{\phi_{1},\phi_{3}\right\}$. The situation is analogous to the computation of the previous marginal, with the difference that now the symbolic combination to carry out is $\phi_{1}\cdot \phi_{3}$. The repositories status after k=3 is shown in Table 3. We have that the third desired marginal is $\psi_{3}=\phi_{5}$.

Finally, for k=4 we have to compute the marginal for $Y=\{X_{2},X_{3}\}$. The relevant potentials are now $\Phi_{Y}=\left\{\phi_{1},\phi_{2},\phi_{3}\right\}$. Variable $X_{1}$ has to be deleted from this set of potentials. As all the potentials contain this variable, as a first step it is necessary to combine all of them, and afterwards to remove $X_{1}$ by marginalizing on $\left\{X_{2},X_{3}\right\}$. Assume that the order of the symbolic operations is: first combine $\phi_{1}$ and $\phi_{2}$ and then its result is combined with $\phi_{3}$; then this result is marginalized by removing $X_{1}$. Then the repositories after k=4 are as presented in Table 4. The combination of $\phi_{1}$ and $\phi_{2}$ was previously carried out for k=2 and therefore its result can be retrieved without new computations.

After that, the numerical part of operations in Table 4 are carried out in the same order in which they are described in that table. In this process, after doing an operation with an identifier (id) equal to *t*, the potentials with time equal to *t* are removed from the $R_{\Phi }$ table. For example, in this case, potentials $\phi_{2}$ and $\phi_{3}$ can be removed from $R_{\Phi }$ after doing operation with id=4 and potential $\phi_{6}$ can be removed after operation with id=5, leaving only in $R_{\Phi }$ the potentials containing the desired marginal potentials needed to compute the KL divergence between both networks (potentials with time=−1).

## 5. Experiments

In order to compare the computation approaches presented in the paper the experimentation uses a set of Bayesian networks available in the *bnlearn* [19] repository (https://www.bnlearn.com/bnrepository/, accessed on 24 August 2021). This library provides all the functions required for the process described below. Given a certain Bayesian network as defined in the repository:

- A dataset is generated using the *rbn* function. As explained in the library documentation, this function simulates random samples from a Bayesian network, using *forward/backward* sampling.
- The dataset is used for learning a Bayesian network. For this step, the *tabu* function is employed using the default setting (a dataset as unique argument). It is one of the structural learning methods available on *bnlearn*. Since the learned model could have unoriented links, the *cextend* function is required, which results in a Bayesian network consistent with the model passed as argument. Any other different learning algorithm could have been used, since the goal is to have an alternative Bayesian network that will be used later to calculate the Kullback–Leibler divergence with the methods described in Algorithms 1 and 2.

For each network, the Kullback–Leibler divergence is computed with the procedures presented using evidence propagation (see Algorithm 1) and using operations cache (described in Algorithm 2). The main purpose of the experiment is to get an estimation of the computation times required for both approaches. The obtained results are included in Table 5. It contains the following information:

- **Network** name.
- Number of **nodes**.
- Number of **arcs**.
- Number of **parameters** required for quantifying the uncertainty of the network.
- **time1**: Runtime using the algorithm without cache (Algorithm 1).
- **time2**: Runtime using the algorithm with cache (Algorithm 2).
- **ops**: Number of elementary operations stored in the operations repository $R_{O}$ to compute all the necessary distributions for the calculation using Algorithm 2.
- **rep**: Number of operations that are repeated and that, thanks to the use of $R_{O}$ and $R_{\Phi }$, will be executed only once.
- **del**: Number of factors that were removed from the $R_{\Phi }$ with the consequent release of memory space for future calculations.

The experiments have been run in a desktop computer with an Intel(R) Xeon(R) Gold 6230 CPU working at 3.60GHz (80 cores). It has 312 Gb of RAM memory. The operating system is Linux Fedora Core 34.

It is observed that the calculation with the second method always offers shorter runtimes than the first one. The shortest runtimes are presented in the table with bold style. It is noteworthy that the case of three networks in which the method based on the use of evidence cannot be completed because the available memory capacity is exceeded: *water*, *mildew*, and *barley*. Moreover, the computational overhead required to manage operations and factor repositories is beneficial as it avoids the repetition of a significant number of operations and enables unnecessary potentials to be released, especially in the most complex networks.

## 6. Conclusions

Computing the KL divergence between the joint probabilities associated with two Bayesian networks is an important task that is relevant for many problems, for example assessing the accuracy of Bayesian network learning algorithms. However, in general, it is not possible to find this function implemented in software packages for probabilistic graphical models. In this paper, we provide an algorithm that uses local computation to calculate the KL divergence between two Bayesian networks. The algorithm is based in a procedure with two stages. The first one plans the operations determining the repeated operations and the times in which potentials are no longer necessary, while the second one carries out the numerical operations, taking care to reuse the results of repeated operations instead of repeating them and deallocating the memory space associated with useless potentials. Experiments show that this strategy saves time and space, especially in complex networks.

The functions have been implemented in Python taking as basis *pgmpy* software package and are available in the *github* repository: https://github.com/mgomez-olmedo/KL-pgmpy, accessed on 24 August 2021. The *README* file of the project offers details about the implementation and the methods available for reproducing the experiments.

In the future, we plan to further improve the efficiency of the algorithms. The main line will be to invest more time in the planning stage looking for deletion orderings minimizing the total number of operations or optimizing the order of combinations, when several potentials have to be multiplied.

## Figures and Tables

**Table 1:** Initial state for $R_{\Phi }$ (left part) and $R_{O}$ (right part).

Rep. of Potentials ($R_{\Phi }$)			Rep. of Operations ($R_{O}$)				
Potential	Time		Id	Type	Arg. 1	Arg. 2	Result
$\phi_{1}\left(X_{1}\right)$	0						
$\phi_{2}\left(X_{1},X_{2}\right)$	0						
$\phi_{3}\left(X_{1},X_{3}\right)$	0						

**Table 2:** Repositories after k=2.

Rep. of Potentials ($R_{\Phi }$)			Rep. of Operations ($R_{O}$)				
Potential	Time		Id	Type	Arg. 1	Arg. 2	Result
$\phi_{1}\left(X_{1}\right)$	−1		1	Comb	$\phi_{1}$	$\phi_{2}$	$\phi_{4}$
$\phi_{2}\left(X_{1},X_{2}\right)$	1						
$\phi_{3}\left(X_{1},X_{3}\right)$	0						
$\phi_{4}\left(X_{1},X_{2}\right)$	−1						

**Table 3:** Repositories after k=3.

Rep. of Potentials ($R_{\Phi }$)			Rep. of Operations ($R_{O}$)				
Potential	Time		Id	Type	Arg. 1	Arg. 2	Result
$\phi_{1}\left(X_{1}\right)$	−1		1	Comb	$\phi_{1}$	$\phi_{2}$	$\phi_{4}$
$\phi_{2}\left(X_{1},X_{2}\right)$	1		2	Comb	$\phi_{1}$	$\phi_{3}$	$\phi_{5}$
$\phi_{3}\left(X_{1},X_{3}\right)$	2						
$\phi_{4}\left(X_{1},X_{2}\right)$	−1						
$\phi_{5}\left(X_{1},X_{3}\right)$	−1						

**Table 4:** Repositories after k=4.

Rep. of Potentials ($R_{\Phi }$)			Rep. of Operations ($R_{O}$)				
Potential	Time		Id	Type	Arg. 1	Arg. 2	Result
$\phi_{1}\left(X_{1}\right)$	−1		1	Comb	$\phi_{1}$	$\phi_{2}$	$\phi_{4}$
$\phi_{2}\left(X_{1},X_{2}\right)$	4		2	Comb	$\phi_{1}$	$\phi_{3}$	$\phi_{5}$
$\phi_{3}\left(X_{1},X_{3}\right)$	4		3	Comb	$\phi_{1}$	$\phi_{3}$	$\phi_{5}$
$\phi_{4}\left(X_{1},X_{2}\right)$	−1		4	Comb	$\phi_{4}$	$\phi_{3}$	$\phi_{6}$
$\phi_{5}\left(X_{1},X_{3}\right)$	−1		5	Marg	$\phi_{6}$	1	$\phi_{7}$
$\phi_{6}\left(X_{1},X_{2},X_{3}\right)$	5						
$\phi_{7}\left(X_{2},X_{3}\right)$	−1						

**Table 5:** Runtimes for KL computation without cache (time1) and with cache (time2).

Network	Nodes	Arcs	Parameters	Time1	Time2	Ops	Rep	Del
cancer	5	4	18	0.0313	**0.012**	48	21	13
earthquake	5	4	10	0.0316	**0.0091**	31	15	5
survey	5	6	21	0.0504	**0.0143**	49	23	12
asia	8	8	18	0.0787	**0.0173**	62	28	13
sachs	11	17	178	0.3484	**0.0409**	81	30	25
child	20	25	230	0.8796	**0.0726**	142	58	56
insurance	27	52	984	16.9990	**0.3788**	631	313	223
water	32	66	10,083	–	**8.6822**	640	299	170
mildew	35	46	540,150	–	**15.9393**	1278	955	150
alarm	37	46	509	4.0949	**0.3354**	638	415	132
barley	48	84	114,005	–	**205.6597**	2273	1695	328
hailfinder	56	66	2656	362.5262	**0.8498**	1197	921	127
hepar2	70	123	1453	23.1047	**1.4088**	1864	1459	242
win95pts	76	112	2656	404.4568	**1.0080**	960	458	314

## Data Availability

Not applicable.
